# Economic Cost of Rehabilitation with Robotic and Virtual Reality Systems in People with Neurological Disorders: A Systematic Review

**DOI:** 10.3390/jcm13061531

**Published:** 2024-03-07

**Authors:** Roberto Cano-de-la-Cuerda, Aitor Blázquez-Fernández, Selena Marcos-Antón, Patricia Sánchez-Herrera-Baeza, Pilar Fernández-González, Susana Collado-Vázquez, Carmen Jiménez-Antona, Sofía Laguarta-Val

**Affiliations:** 1 Department of Physical Therapy, Occupational Therapy, Rehabilitation and Physical Medicine, Faculty of Health Sciences, Universidad Rey Juan Carlos, 28922 Madrid, Spain; roberto.cano@urjc.es (R.C.-d.-l.-C.); patricia.sanchezherrera@urjc.es (P.S.-H.-B.); pilar.fernandez@urjc.es (P.F.-G.); susana.collado@urjc.es (S.C.-V.); sofia.laguarta@urjc.es (S.L.-V.); 2 Multiple Sclerosis Association of Leganés (ALEM), 28915 Leganés, Spain; aitorblazquezfernandez@outlook.es

**Keywords:** cost minimization, cost-effectiveness, cost utility, cost benefit, economic cost, neurological disorders, robotic, virtual reality

## Abstract

**Background**: The prevalence of neurological disorders is increasing worldwide. In recent decades, the conventional rehabilitation for people with neurological disorders has been often reinforced with the use of technological devices (robots and virtual reality). The aim of this systematic review was to identify the evidence on the economic cost of rehabilitation with robotic and virtual reality devices for people with neurological disorders through a review of the scientific publications over the last 15 years. **Methods**: A systematic review was conducted on partial economic evaluations (cost description, cost analysis, description of costs and results) and complete (cost minimization, cost-effectiveness, cost utility and cost benefit) studies. The Preferred Reporting Items for Systematic Reviews and Meta-Analyses (PRISMA) guidelines were followed. The main data sources used were PubMed, Scopus and Web of Science (WOS). Studies published in English over the last 15 years were considered for inclusion in this review, regardless of the type of neurological disorder. The critical appraisal instrument from the Joanna Briggs Institute for economic evaluation and the Consolidated Health Economic Evaluation Reporting Standards (CHEERS) were used to analyse the methodological quality of all the included papers. **Results**: A total of 15 studies were included in this review. Ten papers were focused on robotics and five on virtual reality. Most of the studies were focused on people who experienced a stroke. The robotic device most frequently used in the papers included was InMotion^®^ (Bionik Co., Watertown, MA, USA), and for those focused on virtual reality, all papers included used semi-immersive virtual reality systems, with commercial video game consoles (Nintendo Wii^®^ (Nintendo Co., Ltd., Kyoto, Japan) and Kinect^®^ (Microsoft Inc., Redmond, WA, USA)) being used the most. The included studies mainly presented cost minimization outcomes and a general description of costs per intervention, and there were disparities in terms of population, setting, device, protocol and the economic cost outcomes evaluated. Overall, the methodological quality of the included studies was of a moderate level. **Conclusions**: There is controversy about using robotics in people with neurological disorders in a rehabilitation context in terms of cost minimization, cost-effectiveness, cost utility and cost benefits. Semi-immersive virtual reality devices could involve savings (mainly derived from the low prices of the systems analysed and transportation services if they are applied through telerehabilitation programmes) compared to in-clinic interventions.

## 1. Introduction

The prevalence of neurological disorders, a complex set of conditions resulting from disease of or injury to the nervous system, is increasing around the world. It is estimated that up to one billion people worldwide are affected by neurological disorders, constituting 6.3% of the global disease burden [1].

Neurorehabilitation is understood as a process aimed at reducing the impairment, activity limitation and participation restriction experienced by individuals because of a neurological disease. The professionals involved in this field aim to reduce the degree of functional impairment in patients. It should be understood as an educational and dynamic process based on the adaptation of the individual and their environment to neurological deterioration [2]. The World Health Organization (WHO) defined the term neurorehabilitation as “an active process through which disabled individuals due to neurological injury or disease achieve complete recovery or, if not possible, can optimize their physical, mental, and social potential and integrate into the most appropriate environment” [3].

In recent decades, the conventional rehabilitation for people affected by a neurological disorder has often been integrated with the use of technological devices [4]. In fact, recovery has been shown to depend on the intensity of the therapy and repetition of functional movements, along with performance-dependent feedback and developing motivation among patients during the process [5]. These are the main reasons for proposing the use of these technological devices, such as robots and virtual reality systems, to promote experience-dependent neural plasticity as the basis for motor learning.

Robots used for rehabilitation purposes are classified in terms of (a) the body function that they aim to rehabilitate or in terms of their design (robots for upper limbs versus lower limbs), with a subdivision for the side of the body treated (bilateral versus unilateral robots) and (b) design (exoskeletons, end-effector, or hybrid robots; there are also two kinds of exoskeletons, grounded exoskeletons, which allow walking on a treadmill, and overground wearable exoskeletons) [6]. On the other hand, virtual reality systems are classified as immersive (systems that include projection onto a concave surface or a head-mounted display), semi-immersive (normally related to a single screen projection) and non-immersive (e.g., using a desktop, joysticks or pad displays), with different degrees of immersion and interaction among each of them [7].

There are many advantages derived from using robots and virtual reality systems in neurorehabilitation. These advantages are mainly related to the increase in intensity, number of repetitions, specificity and feedback during rehabilitation [8]. These devices, widely present in specialized rehabilitation centres with a high number of patients with neurological disorders, are considered helpful for assessing deficits and hence for assessing rehabilitation outcomes; they are also treatment tools, which are managed by specialized and trained personnel. However, there are barriers to their adoption [8,9]. Although the scientific literature appears to provide strong support for certain technology-based approaches, their rate of adoption lags far behind the rate that might be expected considering the potential positive consequences associated with their use and the supporting scientific evidence. Some of the reasons for this are related to scientific ignorance, the population and the market to which they are directed, the need for specialized training, ethical aspects, the organization of neurorehabilitation services, technological limitations and challenges, and economic costs [10,11].

Several economic barriers to the adoption of robots and/or virtual reality in neurorehabilitation have been described [11], mainly related to their cost given the difference between them and the different device subtypes. In a context where healthcare costs are continuously rising, there are serious concerns about the economic sustainability of the system, particularly for chronic illnesses such as neurological disorders susceptible to neurorehabilitation where the effectiveness of a new technology is a necessary but not sufficient condition for its adoption. To date, detailed and rigorous studies on the economic cost and/or economic sustainability of these technologies for neurorehabilitation have been very sporadic [12].

Economic studies in health sciences may offer useful information for decision-makers at different levels (e.g., political, economic, care) to treat a specific phenomenon because they might offer clear recommendations about the efficiency of using health resources and the best alternatives. This may be achieved completely (cost minimization, cost-effectiveness, cost utility and cost–benefit analysis) or partially (cost description, cost analysis, description of costs and results), with the former allowing us to compare the effectiveness and costs of at least two interventions, while the latter can address these components independently (Table 1) [13,14]. To the best of our knowledge, no prior systematic review has been conducted that analysed the economic cost related to robots and virtual reality devices in people with neurological disorders in a rehabilitation context. 

Accordingly, the aim of this systematic review was to identify the evidence on the economic cost of rehabilitation with robotic and virtual reality devices for people with neurological disorders in a rehabilitation context through a review of scientific publications over the last 15 years.

## 2. Methods

### 2.1. Design

A systematic review was conducted on partial economic evaluations (cost description, cost analysis, description of costs and results) and complete studies (cost minimization, cost-effectiveness, cost utility and cost benefit). The Preferred Reporting Items for Systematic Reviews and Meta-Analyses (PRISMA) [15] guidelines were used to carry out this systematic review starting with a PICORT (patient/population, intervention, comparison, outcomes, resources, and time horizon) question.

Population: People with neurological disorders without restrictions on the type of neurological disorder, age, sex, time from injury (if applicable) or severity.

Intervention: Any type of intervention using robots and/or virtual reality devices whose objective was to improve motor impairments.

Comparison: Conventional rehabilitation therapy, other rehabilitation approaches, usual care or no treatment.

Outcomes: Cost minimization, cost-effectiveness (independent of the scale but related to motor impairment outcomes), cost utility, cost benefit, cost analysis or a description of costs.

Resources: Cost of the intervention (if it was available), which was understood as the value of the resources used to provide a service or perform an intervention, according to the perspective taken in the study, with denomination of type of currency and current year. 

Time horizon: Time period reported in each study.

This systematic review was registered in PROSPERO prior to its execution under reference number CRD42023461806.

### 2.2. Search Strategy

A systematic comprehensive literature search was conducted from August to October 2023 to identify original studies that answered the PICORT question, using the following data sources: PubMed, Scopus and Web of Science (WOS). After identifying eligible articles, a cross-search of their references was also completed for additional studies.

The detailed search strategy for each database is shown in Table 2. The search strategy consisted of controlled vocabulary and primary keywords and different combinations of Boolean operators. The keywords included stroke, traumatic brain damage, spinal cord injury, multiple sclerosis, Parkinson’s disease, cerebral palsy, cost minimization, cost-effectiveness, cost utility, cost benefit, economic cost, neurological disorders, and robotic and virtual reality, among others. For a detailed description of the search strategy, see Table 2.

Two authors independently searched and screened titles and abstracts to identify studies meeting the inclusion criteria. Duplicates were removed and disagreements regarding the selection of studies were resolved by a third author.

### 2.3. Inclusion and Exclusion Criteria

Studies published in English over the last 15 years were considered for inclusion in this review, regardless of their methodological design. Published papers were also included in the systematic review regardless of the type of neurological disorder, age, sex, time from injury (if applicable) and severity. Studies were included if the papers evaluated the economic cost of rehabilitation using robotic and/or virtual reality devices, notwithstanding their classification or type. We applied no restrictions on the rehabilitation settings (e.g., hospitals, outpatient rehabilitation clinics). This review considered studies that had the following outcomes: cost minimization, cost-effectiveness (independent of the scale but related to motor impairment outcomes), cost utility, cost benefit, cost analysis or a description of costs.

We included studies with any type of intervention using robots and/or virtual reality devices whose objective was to improve motor impairments, which was compared with conventional rehabilitation therapy, other rehabilitation approaches, usual care or no treatment. We considered eligible multi-session studies that performed treatments with various durations, intensities and frequencies with time-dependent clinical follow-up.

The exclusion criteria were as follows: study protocols, poster communications, contributions to congresses or symposium reports, and studies without information about economic cost related to robots and/or virtual reality devices in people with neurological disorders for neurorehabilitation purposes.

### 2.4. Data Extraction

The following data were extracted from the papers: authors, country, type of economic cost studied, disease, sample, technology used, dosage, currency, data analysis and authors’ conclusions on the cost comparisons.

The authors independently collected these data and eventually reached a consensus on the extracted data, resolving disagreements through discussion with a third reviewer.

Given the high heterogeneity expected in terms of the devices used, outcome measures, intervention modalities and comparator(s) analysed, a narrative description of the collected results was planned.

### 2.5. Methodological Quality

The selected studies were critically appraised by two independent reviewers for their methodological quality using the standardized critical appraisal instrument from the Joanna Briggs Institute for economic evaluation [16]. Disagreements that arose between the reviewers were resolved through a third reviewer. All studies regardless of their methodological quality underwent data extraction and synthesis to maximize the data collection.

In addition, the studies were assessed using the Consolidated Health Economic Evaluation Reporting Standards (CHEERS) [17]. CHEERS is made up of 28 items and is primarily designed for reporting economic studies in scientific journals, and it is helpful for researchers in the planning stage of economic studies and for Health Technology Assessment agencies, given the increasing emphasis on transparency in decision-making processes. The percentage of the CHEERS criteria that was met by each included study was determined.

## 3. Results

**Figure 1:**
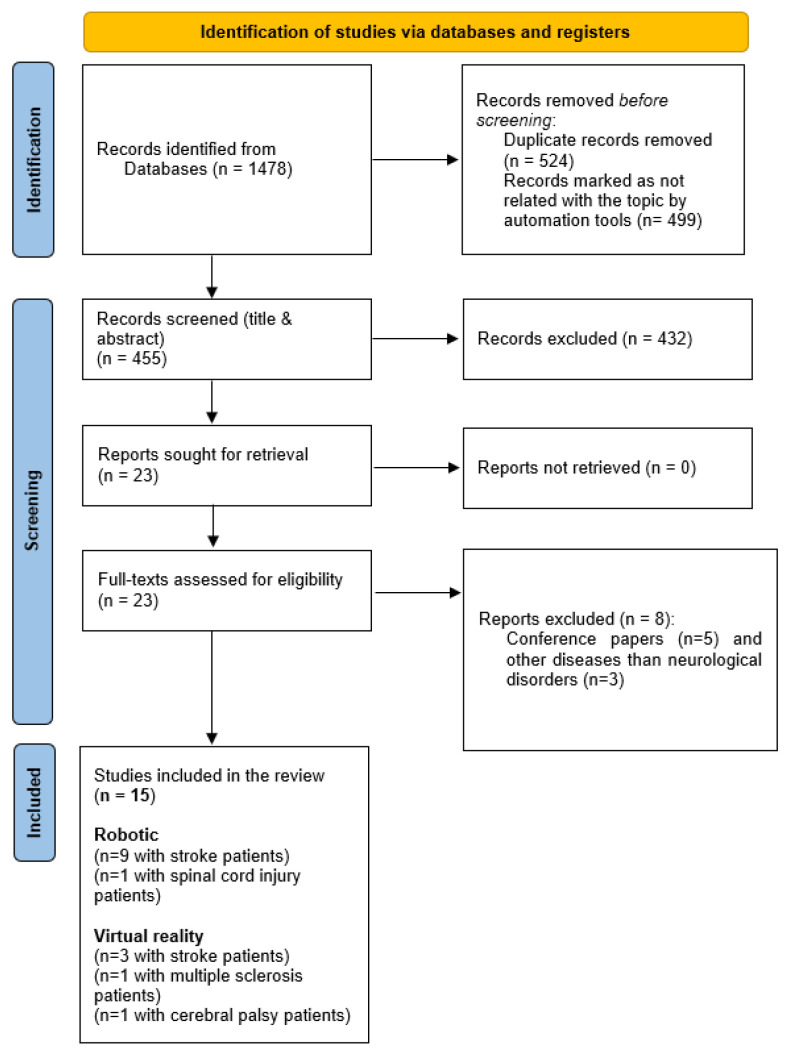
Flow chart of the identified studies according to the PRISMA 2020 Statement.

A total of 1478 papers were initially found from the database searches, of which, 1023 records were removed before screening mainly due to being duplicates. After the initial screening of titles and abstracts, 432 records were excluded due to not fulfilling the inclusion criteria. A total of 23 records were assessed for eligibility, with 8 being excluded for different reasons (conference papers and studied diseases other than neurological disorders). Finally, 15 studies [18,19,20,21,22,23,24,25,26,27,28,29,30,31,32] were included in the systematic review and were appraised for quality (Figure 1).

### 3.1. Characteristics of Included Studies

**Figure 2:**
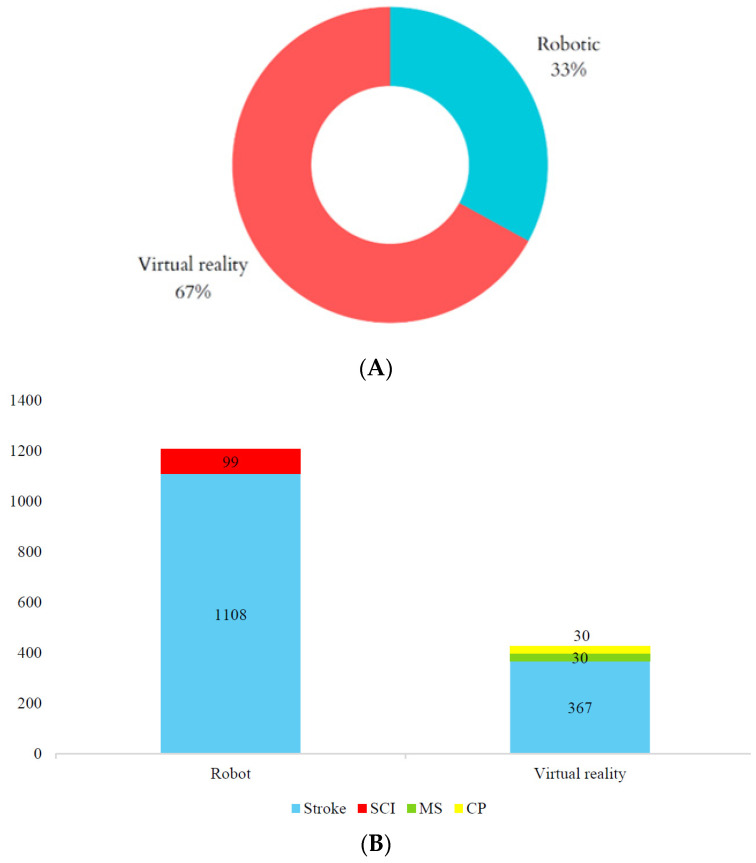
Graphical distribution of included papers. (**A**) Percentage distribution of studies by intervention. (**B**) Number of patients in robotics and virtual reality studies. SCI: spinal cord injury; MS: multiple sclerosis; CP: cerebral palsy.

A total of 15 studies with 1634 patients were included in this review (1108 individuals who experienced a stroke and 99 with a spinal cord injury for robotic studies; 367 people who experienced a stroke, 30 with multiple sclerosis and 30 with cerebral palsy for virtual reality studies). Ten papers were focused on robotics [18,19,20,21,22,23,24,25,26,27] and five on virtual reality [28,29,30,31,32] (Figure 2). The studies showed a greater predominance of men than women.

Regarding the papers focused on robotics, nine records recruited people who experienced a stroke [18,20,21,22,23,24,25,26,27] and one record recruited people with an SCI [19]. Six studies employed robots for upper-limb rehabilitation [18,20,21,22,25,26], two for lower-limb rehabilitation [24,27], and two for upper- and lower-limb rehabilitation [19,23]. Two papers [18,19] used a combination of robots for their aims. The robotic device most frequently used for the upper limb was InMotion^®^ (Bionik Co., Watertown, MA, USA) [20,21,25,26], followed by (with an equal number) NeReBot^®^ (University of Padua, Padua, Italy) [22], the Theradrive system^®^ (University of Pennsylvania, PA, USA) [19] and a combination of Motomed Viva 2^®^ (MOTOmed ©, Betzenweiler, Alemania), Bi-ManuTrack^®^ (Reha-Stim Inc., Berlin, Germany), RehaDigit^®^ (HASOMED, Magdeburg, Germany), Reha-Slide^®^ and Reha-Slide duo^®^ (Reha-Stim Medtec Inc., New York, NY, USA) [18], and Hand Mentor^®^ (Motus Nova Inc, Atlanta, GA, USA) [23]. For lower-limb rehabilitation, Foot Mentor^®^ (Motus Nova Inc., Atlanta, GA, USA) [23], Robert^®^ (Life Science Robotics Inc, Aalborg, Dinamarca) [24], Motomed Viva 2^®^ for lower extremities [19] and an undeclared robot in [27] were used in equal numbers.

**Figure 3:**
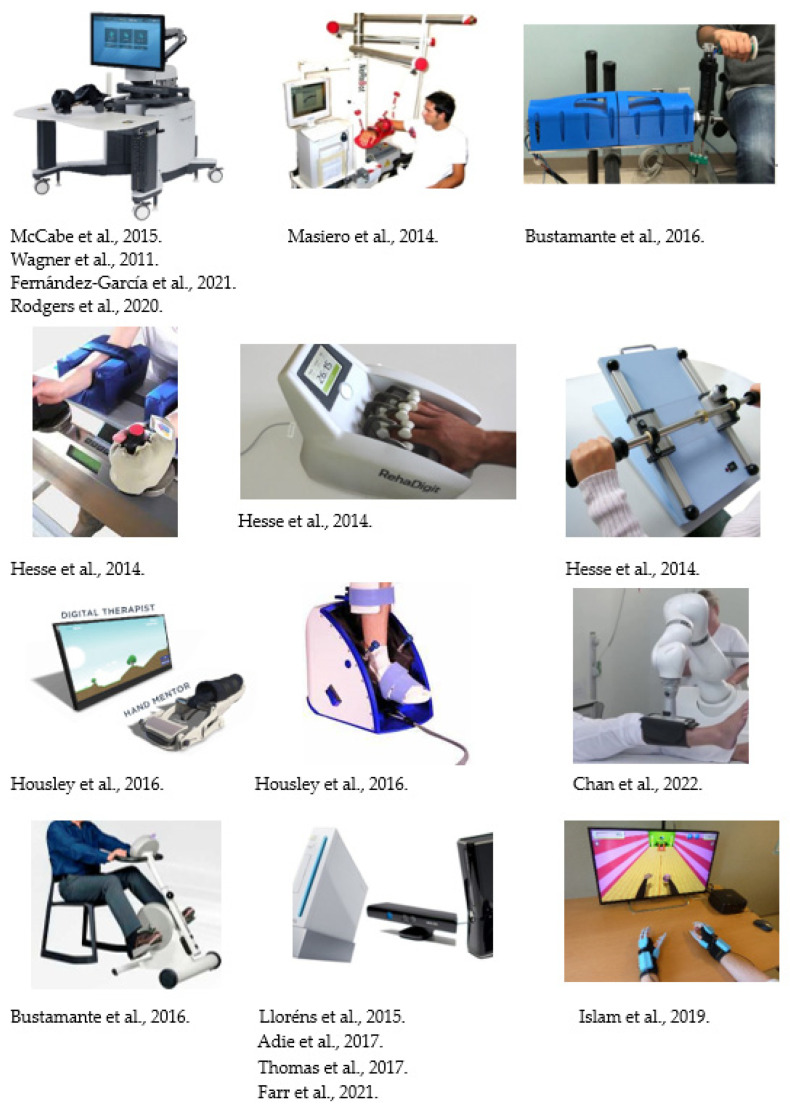
Technological devices employed in the included studies [18,19,20,21,22,23,24,25,26,28,29,30,31,32].

Regarding the studies focused on virtual reality, three papers recruited people who experienced a stroke [28,29,30], one research recruited people with multiple sclerosis [31] and one paper recruited people with cerebral palsy [32] (Figure 2). Two papers investigating economic costs were focused on upper-limb rehabilitation [29,30], two on lower-limb rehabilitation [28,32], and one on upper- and lower-limb rehabilitation [31] with virtual reality. All included papers used semi-immersive virtual reality systems. Four papers [28,30,31,32] used commercial video game consoles (Nintendo Wii^®^ (Nintendo Co., Ltd., Kyoto, Japan) and Kinect^®^ (Microsoft Inc., Redmond, WA, USA)), while Islam et al. [29] used Bi-Manu-Trainer^®^ (Figure 3).

Patients were recruited in different phases of stroke recovery (subacute and chronic phases), with a greater predominance of chronic patients. Patients with a spinal cord injury (SCI) were recruited in the acute and chronic phases [27]. There was no description of the Expanded Disability Status Scale (EDSS) for the recruited multiple sclerosis patients [31]. Patients with a Gross Motor Function Classification System (GMFCS) level I or II impairment were recruited in [32]. The clinical characteristics of the included studies, technology used, targeted body part and protocol used are shown in Table 3.

**Figure 4:**
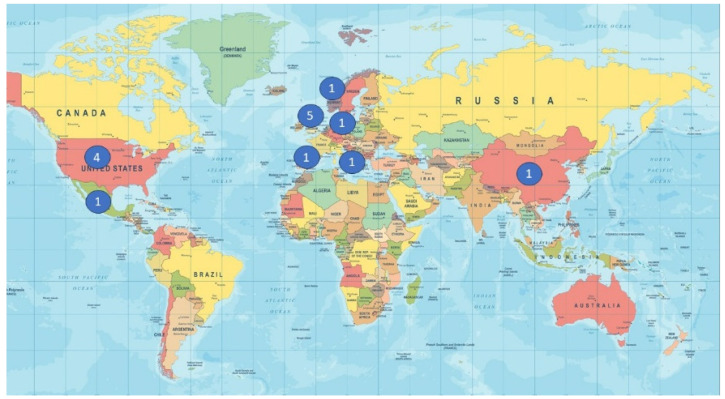
Geographical distribution of economic evaluation studies.

The studies came from various countries (Figure 4), namely the United States of America [20,21,23,27], Mexico [19], Spain [28], Germany [18], Italy [22], Denmark, Norway and Belgium (a multicentric international study) [29], the United Kingdom [25,26,30,31,32] and China [24].

Most of the included papers related to robotic devices were conducted in a clinical setting [18,19,20,21,22,24,25,26,27], apart from Housley et al. (2016) [23], which was conducted at the participants’ homes. Most studies related to virtual reality were conducted at the participants’ homes [30,31,32], while one was in a clinical setting [29] and one compared between performing the intervention in participants’ homes and a clinical setting [28].

The trial with the largest sample size [25,26] was conducted with people who experienced a stroke undergoing upper-limb rehabilitation with a robotic device. Specifically, in these papers [25,26], 257 people who experienced a stroke received robot-assisted training plus usual care, 259 underwent an enhanced upper-limb therapy programme plus usual care and 254 received the usual care. The trials with the lowest sample size were those conducted by Bustamante et al. [19] and Housley et al. [23], with 20 stroke patients each. In Bustamante et al. [19], ten subjects received traditional therapy and the rest of them received a combination of robots (Robot Gym) for upper-limb and lower-limb rehabilitation. In Housley et al. [23], ten people who experienced a stroke received rehabilitation for the upper limb with a robot (Hand Mentor^®^) and the other ten subjects received lower-limb rehabilitation with Foot Mentor^®^.

The studies used different training durations, with the time per session ranging from 30 [18] to 300 min [20] (Table 3). The longest duration was 90 days [23] and the shortest was 16 days [29]. Several studies did not report the dosage of the intervention or it was not reported clearly [22,24,27,31,32].

There were different types of comparisons in the studies included in this systematic review on robotic interventions, which included comparisons between a robotic intervention and a conventional rehabilitation approach and/or usual care [19,21], comparisons between a robotic intervention plus conventional rehabilitation and dose-matched usual care and conventional approaches [18,20,25,26], and comparison between a robotic upper-limb intervention and a robotic lower-limb intervention [23]. All these aforementioned studies were focused on people who experienced a stroke. The only study focused on people with a spinal cord injury, which was conducted by Pinto et al. [27], compared conventional training and overground robotic training.

Regarding the studies focused on virtual reality, one paper [28] compared in-clinic rehabilitation with virtual reality and an at-home intervention using virtual reality in people who experienced a stroke. Islam et al. [29] and Adie et al. [30] compared an intervention using virtual reality and a conventional rehabilitation in people with stroke. Thomas et al. [31] compared a Nintendo Wii plus usual care intervention and usual care in people with multiple sclerosis. Finally, Farr et al. [32] compared a supervised and a unsupervised virtual reality group.

The included studies mainly presented cost minimization outcomes and a general description of costs per intervention (Table 3), with Garcia et al. [25] and Rodgers et al. [26] being the studies that investigated cost minimization, cost-effectiveness and cost utility. The cost per patient, depending on the type of intervention, the device used, the duration of the study design and the country, varied among the included studies. The currency and cost data derived from the experimental and control treatments are shown in Table 4.

The main economic conclusions drawn by the authors and the recommendations derived from each study are also shown in Table 4. Hesse et al. [18], Bustamante-Valles [19], Wagner et al. [21], Masiero et al. [22], Housley et al. [23] and Chan et al. [24] showed that robotic interventions, despite differences in their protocols, might present more advantages than traditional therapy in terms of economic cost in people who experienced a stroke. However, McCabe et al. [20], Fernandez-Garcia et al. [25] and Rodgers et al. [26] did not report cost-effectiveness, but the conventional interventions were less expensive than robotics for people who experienced a stroke. Finally, Pinto et al. [27] showed that the most cost-effective locomotor training strategy for people with an SCI differed depending on injury completeness (Table 3 and Table 4).

The results related to virtual reality interventions showed that semi-immersive virtual reality devices could involve savings (mainly derived from the low prices of the systems analysed and transportation services if they are applied through telerehabilitation programmes) compared to in-clinic interventions in people who experienced a stroke [28]. However, Islam et al. [29] showed equal improvements for conventional approaches in people with stroke for upper-limb rehabilitation, while Adie et al. [30] did not find such improvements, and the virtual reality intervention was more expensive than the conventional upper-limb rehabilitation in people who experienced a stroke. Thomas et al. [31] and Farr et al. [32] showed the advantages derived from using semi-immersive virtual device systems in people with multiple sclerosis and cerebral palsy (Table 3 and Table 4).

### 3.2. Methodological Quality

Using the critical appraisal instrument from the Joanna Briggs Institute for economic evaluation, the methodological quality scores were calculated and are shown in Table 5. Overall, the methodological quality of the included studies was of a moderate level. The studies presented different scores, ranging from 2 to 10. The average total score was 9.27/11 points (6.88/11 points was the average score for the papers related to robotics with stroke patients [18,19,20,21,22,23,24,25,26]; 7/11 points for the paper related to robotics with spinal cord injury patients [27]; 8/11 points for the papers related to virtual reality with stroke patients [28,29,30]; 4/11 points for the paper related to virtual reality with multiple sclerosis patients [31]; and finally 5/11 points for the paper related to virtual reality with cerebral palsy patients [32]). The paper with the highest methodological quality, based on this critical appraisal instrument, was Wagner et al. [21]. The research with the lowest score was Chan et al. [24].

Considering the questions that comprise the critical appraisal instrument from the Joanna Briggs Institute for economic evaluation, most of the studies showed quality deficiencies from question 7 to 11 (Q7: Are costs and outcomes adjusted for differential timing? Q8: Is there an incremental analysis of costs and consequences? Q9: Were sensitivity analyses conducted to investigate uncertainty in estimates of cost or consequences? Q10: Do study results include all issues of concern to users? Q11: Are the results generalizable to the setting of interest in the review?). The total scores from each question are shown in Table 5 and ranged from 93.33% to 20%.

**Figure 5:**
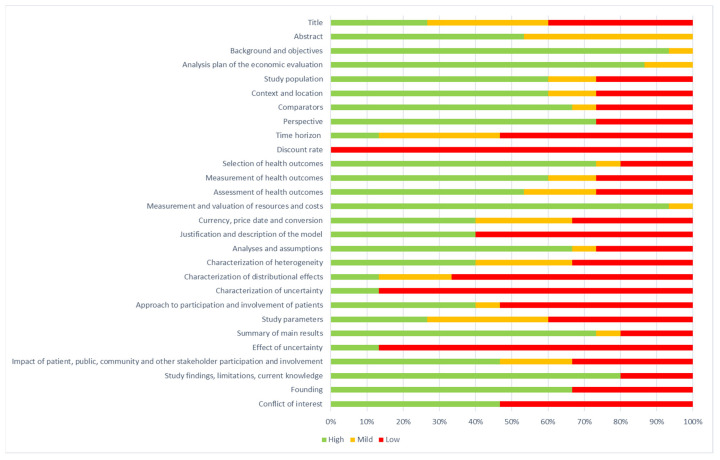
Consolidated Health Economic Guide Reporting Standards (CHEERS) scores.

The information about the CHEERS scores is presented in Figure 5. The items with the best scores were “background and objectives”, “measurements and evaluation of resources and cost” and “analysis plan of the evaluation”. The items with the lowest scores were “discount rate”, “characterization of uncertainty” and “effect of uncertainty”. Overall, the CHEERS evaluation of the included papers showed medium scores. None of the items showed a high score for all of the papers. All papers achieved a low score for the “discount rate” item.

## 4. Discussion

### 4.1. General Considerations of the Included Articles

The objective of this research was to identify the evidence on the economic cost of rehabilitation using technology (robotic and virtual reality devices) for people with neurological disorders. The first finding of this work was the limited number of studies focused on this topic. It is striking that of the 15 papers included in the present systematic review, 10 of them were about robotics (9 focused on patients who experienced a stroke and 1 on people with an SCI). Five of the included papers focused on the use of virtual reality (three in people who experienced a stroke, one in people with multiple sclerosis and one in people with cerebral palsy). Despite the epidemiological reality, there are no economic data on robotics and virtual reality in people with Parkinson’s disease, Alzheimer’s disease, polyneuropathies and peripheral neuropathies or muscular dystrophies, for example.

The use of robotic devices has been the most widely studied in the paper included in our systematic review. Economic studies about the use of robotics for the upper limbs with the MIT-Manus robotic system (InMotion^®^ commercial version) were the most common. Other works focused on upper-limb rehabilitation with robotics using NeReBot^®^, a combined group of robots (called Robot Gym by the authors) or robot-assisted group therapy (Bi-ManuTrack^®^, RehaDigit^®^, Reha-Slide^®^ and Reha-Slide duo^®^). No studies that met the search criteria of this review calculated the economic cost of other devices widely used in clinical settings such as Armeo Spring^®^, Armeo Power^®^, AMADEO^®^ or MIME^®^, to name just a few examples. Two works focused on the upper and lower limbs [19,23], another paper used ROBERT^®^ and finally another study did not report the robotic systems used for lower-limb rehabilitation [27]. The presence of works that did not identify the devices used [27] and the lack of data in studies on economic costs are paradigmatic [24].

Among the virtual reality systems used, none of the included studies used immersive systems like head-mounted displays (HMDs) or Cave-Assisted Virtual Environments (CAVE), which require a large financial investment and are a reality in many neurorehabilitation centres. Most of the research on this topic was carried out using commercial video game consoles (Nintendo Wii^®^ and Kinect^®^), except for Islam et al. [29] (with Bi-Manu-Trainer^®^), and were all included under the classification of semi-immersive virtual reality systems.

### 4.2. Economic Cost of Robotics and Virtual Reality in Neurorehabilitation

The scientific literature seems to clearly indicate, for certain neurological disorders, that robot-assisted rehabilitation and virtual reality are effective for functional recovery (including improvement in gait and upper-limb function) for patients who experienced stroke, and patients with traumatic brain injury, spinal cord injuries, cerebral palsy, Parkinson’s disease and multiple sclerosis [33]. Nonetheless, as previously mentioned, the widespread use of these innovative technologies in the neurorehabilitation field is limited by several issues. Among them, economic barriers to the adoption of these technologies are linked to inadequate evaluation and cost-effectiveness studies, reimbursement models and other incentive mechanisms [11].

Currently, detailed and rigorous studies on the economic cost of robotic and virtual reality technologies in neurorehabilitation are scarce. Furthermore, it is worth noting the terminological problems associated with the economic studies included in this systematic review. Although in many cases, the authors carried out an exhaustive examination of the derived costs and compared them with other treatment modalities, the term “cost-effectiveness” was used, in most cases, in an erroneous manner as they addresses cost minimization. Considering the conceptual classification of Lo et al. [14] and the concept of economic cost employed in this review, the main argument against the introduction of technology in rehabilitation is economic considerations. It is often said that treatment with technology is expensive, but according to the findings of our work, there is not enough research in this regard to support this statement: some of it is contradictory and, in most cases, non-existent.

As Calabrò et al. [33] asked, what are we comparing? What does expensive mean? If we compare treatment with the use of robotics to cases that we do not treat, or where they receive the usual care, the treatment with robotics certainly seems to be more expensive. However, several authors do not agree with this affirmation. Chan et al. [24] suggest that the ROBERT^®^ robot is better than physical therapy for performing repetitive exercises for lower limbs, as the physiotherapist’s time can be saved when the robot is being used. Their cost analysis showed that employing ROBERT^®^ is less costly than the equivalent performed by a physiotherapist. Although the capital cost of the robotic system is high, its average hourly operating cost is just one-tenth of the cost of one specialty outpatient session in hospitals in Hong Kong. Housley et al. [23] pointed out that a home-based robotic therapy with Hand and Foot Mentor^®^ reduced costs, while expanding access to a rehabilitation modality for people who would not otherwise have received care. Their analysis revealed an average of USD 2352 (64.97%) in savings compared to clinic-based therapy per stroke survivor. Masiero et al. [22] compared several NeReBot^®^ treatment protocols for the upper limbs, comprising different combinations of robotic and non-robotic exercises, and indicated that robotic technology can be a valuable and economically sustainable aid in the management of post-stroke patient rehabilitation. In the same vein, Wagner et al. [21] showed that the cost of delivering robot therapy for the upper limbs with InMotion^®^ plus intensive therapy was USD 5152 and USD 7382, respectively, and both approaches were significantly more expensive than usual care alone. However, the added cost of delivering robot or intensive comparison therapy was recuperated by lower healthcare use costs compared with those in the usual care group. Finally, the use of robots as a combination of several devices into a robotic gym for the upper limbs was as effective as a double session of individual arm therapy in subacute stroke patients [18], or they could enhance functionality in the upper-extremity tests, similar to the patients in the control group. In the lower extremities, the robotics resulted in more improvement than the traditional therapy, thereby making Robot Gym a more cost- and labour-efficient option for countries with scarce clinical resources and funding [19]. However, there is controversy. McCabe et al. [20] showed that all treatment modalities used in their study were effective in improving upper-limb recovery in stroke patients, but the motor learning approach alone protocol was less expensive than the robotics plus motor learning approach protocol. Finally, in the same vein, the cost-effectiveness analyses of Fernandez-Garcia et al. [25] and Rodgers et al. [26] suggested that neither robot-assisted training with InMotion^®^ nor enhanced upper-limb therapy, as delivered in their trials for people recovering from a stroke, was likely to be cost-effective at any of the cost-per-QALY thresholds considered, with the usual care being most likely to be cost-effective at all the willingness-to-pay values considered in the analysis. Finally, Pinto et al. [27] showed that the most cost-effective locomotor training strategy for people with an SCI differed depending on injury completeness, with costs being lower for conventional training at USD 1758 versus overground robotic training at USD 3952, and lower for those with an incomplete versus complete injury. Taking into account the results of the papers on the economic cost of robotics included in the present review, there was heterogeneity related to the robotic devices used (mostly focused on the upper limbs), the type of patients (all studies but one focused on stroke patients), the protocols implemented, unstudied populations (traumatic brain injury, Parkinson’s disease, multiple sclerosis, cerebral palsy, among others) and the settings studied (at home versus the clinical setting) that prevent an analysis of the external validity of the results.

With respect to the studies focused on virtual reality, all the papers included in this review employed commercial video game consoles (Nintendo Wii^®^ and Kinect^®^), and all of them applied and/or compared the treatment modalities with a telerehabilitation approach using virtual reality. Farr et al. [32] indicated that the use of Nintendo Wii Fit^®^ in children with cerebral palsy at home was inexpensive and acceptable over short periods of around six weeks, costing around GBP 30 or GBP 40 per child. Thomas et al. [31] estimated that the cost of the equipment (Nintendo Wii^®^ console plus peripherals and software) was approximately GBP 300 per unit, with the mean cost of delivering Mii-vitaliSe being GBP 684 per person, making it a profitable tool in a chronic disorder such as multiple sclerosis. Lloréns et al. [28], Islam et al. [29] and Adie et al. [30] conducted their research in people who experienced a stroke. Lloréns et al. [28] indicated that semi-immersive virtual reality-based telerehabilitation interventions with Kinect ^®^ can promote the reacquisition of locomotor skills associated with balance in the same way as in in-clinic interventions, when both are complemented by a conventional therapy programme. This semi-immersive virtual reality-based intervention could involve savings (mainly derived from transportation services) compared to in-clinic interventions. On the other hand, Islam et al. [29] showed that additional upper-extremity VR training with Bi-Manu-Trainer^®^ was equally as effective as additional conventional therapy in the subacute phase after stroke, and no cost savings in favour of VR were found. Finally, Adie et al. [30] pointed out that Wii^®^ was not superior to arm exercises in home-based rehabilitation for stroke survivors with arm weakness, and it was more expensive than arm exercises. Therefore, among the articles included in the present research related to virtual reality, there are no papers that studied the economic viability of immersive virtual reality systems (i.e., HMD and CAVE), and there are no studies in populations with highly prevalent neurological disorders (traumatic brain injury, spinal cord injury, Parkinson’s disease, among others), or in comparison with conventional rehabilitation strategies in clinical settings.

### 4.3. Methodological Quality

The methodological quality of the studies included in this systematic review was moderate. However, different aspects in the different studies limited their quality relative to economic cost. There is a need for future studies showing the costs and outcomes adjusted for differential timing. Further, they must incorporate an incremental analysis of costs and consequences related to the use of robotics and virtual reality in a rehabilitation context for people with neurological disorders. Finally, there is a need in future studies to include all aspects of concern to users, clinicians and developers to make it possible to generalize the findings (external validity).

Economic studies should have larger sample sizes to ensure the validity of both the cost and clinical outcomes. Also, they should employ the correct terminology for economic cost studies throughout the research as terms are often used erroneously. Finally, researchers should also disclose their calculation steps to enable a better understanding of how the values of the cost per patient or cost per patient session measures were calculated to make it possible to extract data of interest and to permit comparisons across studies.

### 4.4. Future Research Lines and Practical Implications of This Systematic Review

In the economic sense, rehabilitation with the use of technology (in this case, mostly related to robotics) has proven to be expensive, and the gap between the cost of robotic and conventional therapies is considerable. It is important to note that there are many devices (robotics and virtual reality devices) that were not studied in the papers included in this systematic review but are widely used in the clinical setting, so future studies are therefore needed in this area. Further, the operational costs, replacement costs, the cost of educating skilled therapists and the cost of a device’s maintenance must be computed [33,34]. Also, several studies included in this review showed that the costs seemed to decrease as the hours of possible robot use increased. The use of robots might be a more economical long-term solution as patients present fewer complications that require a greater demand for therapist time and additional health services. Future studies should be conducted, bearing in mind the methodological limitations indicated, to corroborate these findings.

Various protocols aimed at addressing the topic of this work have been published [35,36,37,38], although the conclusions of some of them are not yet available [38]. To the best of our knowledge, this is the first systematic review to investigate the economic cost of robotics and virtual reality in a neurorehabilitation context. In 2018, Lo et al. [39] published their protocol to calculate the economic cost of robotic rehabilitation for adult stroke patients. Subsequently, their systematic review was published with interesting findings [14]. Their results, based on five papers about robotics (with a total of 213 patients; four papers examined upper-limb interventions, and one study evaluated both upper-limb and lower-limb interventions with people in acute, subacute and chronic stroke phases), showed that robotic therapy had a better economic outcome than conventional therapy. For patients with severe disability from a significant stroke, a moderate dominance of robotic therapy in terms of health benefits was found, and a strong dominance of robotic therapy for cost benefit was found. The key sensitivity factors affecting robotic therapy included the number of patients who could be treated per robotic session and the time the therapists spent with patients during a robotic session. These results are in line with those of the studies included in our systematic review [18,19,21,22,23,24]. Although a large number of works have been included in our review, not all of them seem to point in that direction [20,25,26,27], possibly due to the different designs of the studies, economic variables to be considered, the comparison treatments and the type of neurological disorder. There is a lack of information about the economic costs for other prevalent neurological disorders such as traumatic brain damage, Parkinson’s disease, multiple sclerosis and cerebral palsy, among others, despite these being pathologies in which robotics are being widely used.

To the best of our knowledge, no prior review has reviewed the economic costs of virtual reality systems in a neurorehabilitation context. These systems present important advantages compared to other technologies, namely their portability, ease of use, commercial availability, low cost and non-invasive nature [40,41]. Our results seem to point to cost savings with these commercial semi-immersive systems in stroke patients, although future work is needed to analyse the costs of immersive systems compared to traditional rehabilitation approaches or usual care. In addition, it is necessary to clearly establish the environment in which the research is being carried out and expand the population groups beyond stroke patients.

Finally, other papers were discarded from the present review due to their editorial nature [42], e.g., a letter to the editor [43], a conference report [44] and a clinical case study [45]. These papers highlight the interest in the topic among the international scientific community as well as editors of scientific journals. In fact, the effectiveness of a technological device is less difficult to prove compared to its economic efficiency and sustainability in the short, medium and long term [33]. Thus, future studies on the economic cost of technology in neurorehabilitation should consider the recommendations made in this work.

There are some limitations to this review that are important to highlight. First, our systematic review only included paper over the last 15 years, so we cannot rule out the possibility that previous studies have addressed this issue. Also, due to the heterogeneity of the studies included, economic outcome measures and dosage applied, our results must be interpreted with caution. Also, we only selected articles published in English and the search was limited to specific databases, which may have reduced the number of articles included. In addition, the different degrees of methodological quality of the studies, the heterogeneous samples and missing data of the papers are factors that may limit the correct interpretation of our results. Finally, we cannot extrapolate our results to all patients with neurological disorders, with other objectives and/or with other technological devices.

## 5. Conclusions

Controversy about using robotics in people with neurological disorders in a rehabilitation context in terms of cost minimization, cost-effectiveness, cost utility and cost benefit can be found in the literature. On the other hand, semi-immersive virtual reality-based interventions could involve savings (mainly derived from the low prices of the systems analysed and transportation services if they are applied through telerehabilitation programmes) compared to in-clinic interventions. Future studies should be conducted, taking into consideration the methodological limitations indicated and showing the costs and outcomes adjusted for differential timing, incorporating the incremental analysis of costs and consequences related to the use of robotics and virtual reality in a rehabilitation context in people with neurological disorders.

## Figures and Tables

**Table 1:** Type of economic cost studies in rehabilitation.

**Cost minimization**	Studies comparing the cost of providing rehabilitation with technological devices against the cost of providing conventional therapy.
**Cost effectiveness**	Studies comparing the cost of providing rehabilitation with technological devices against the cost of providing conventional therapy; the outcome is presented as the relative cost to achieve a unit of effect.
**Cost utility**	Studies comparing the cost of providing rehabilitation with technological devices against the cost of providing conventional therapy; the outcome is presented as the relative cost to achieve a unit of utility, which is measured in quality-adjusted life-years (QALY).
**Cost benefit**	Studies comparing the cost of providing rehabilitation with technological devices against the cost of providing conventional therapy; the outcome is presented as the relative cost to achieve a unit of benefit, which is measured in direct and undirect monetary units.

Modified from [14].

**Table 2:** Search strategy.

Database	Search Terms	Returns
**Pubmed**	((robotics [MeSH Terms] OR robot [Text Word] OR robot-assisted training [Text Word] OR electromechanical robot [Text Word] OR virtual reality [MeSH Terms] OR virtual reality-based rehabilitation [Text Word] OR video game [Text Word] OR video console [Text Word] OR technology-assisted therapy [Text Word]) AND (stroke [MeSH Terms] OR stroke [Text Word] OR “brain injury” OR “traumatic brain injury” OR spinal cord injury [MeSh Terms] OR spinal cord injury [Text Word] OR multiple sclerosis [MeSh Terms] OR multiple sclerosis [Text Word] OR Parkinson’s disease [MeSh Terms] OR Parkinson’s disease [Text Word] OR parkinson OR cerebral palsy [MeSh Terms] OR cerebral palsy [Text Word] OR neurological disorders [MeSh Terms] OR neurological disorders [Text Word]) AND (cost minimization [MeSh Terms] OR cost effectiveness [MeSh Terms] OR cost utility [MeSh Terms] OR cost benefit [MeSh Terms] OR cost [MeSh Terms] OR cost-analysis [MeSh Terms] OR economic analysis [MeSh Terms] OR economic evaluation [MeSh Terms]))	40
**Scopus**	(TITLE-ABS-KEY (robotics) OR TITLE-ABS-KEY (robot) OR TITLE-ABS-KEY (“robot-assisted training”) OR TITLE-ABS-KEY (“electromechanical robot”) OR TITLE-ABS-KEY(“virtual reality”) OR TITLE-ABS-KEY (“virtual reality-based rehabilitation”) OR TITLE-ABS-KEY (“video game”) OR ALL (“video console”) OR TITLE-ABS-KEY (“technology-assisted therapy”)) AND (TITLE-ABS-KEY (stroke) OR TITLE-ABS-KEY (“brain injury”) OR TITLE-ABS-KEY (“traumatic brain injury”) OR TITLE-ABS-KEY (“spinal cord injury”) OR TITLE-ABS-KEY (“multiple sclerosis”) OR TITLE-ABS-KEY (“Parkinson’s disease”) OR TITLE-ABS-KEY (parkinson) OR TITLE-ABS-KEY (“cerebral palsy”) OR TITLE-ABS-KEY (“neurological disorders”)) AND (TITLE-ABS-KEY (“cost minimization”) OR TITLE-ABS-KEY (“cost effectiveness”) OR TITLE-ABS-KEY (“cost utility”) OR TITLE-ABS-KEY (“cost benefit”) OR TITLE-ABS-KEY (cost) OR TITLE-ABS-KEY (cost-analysis) OR TITLE-ABS-KEY (“economic analysis”) OR TITLE-ABS-KEY (“economic evaluation”))	1267
**Web of Science**	(TS = (robotics) OR TS = (robot) OR ALL = (“robot-assisted training”) OR ALL = (“electromechanical robot”) OR TS = (“virtual reality”) OR ALL = (“virtual reality-based rehabilitation”) OR ALL = (“video game”) OR ALL = (“video console”) OR ALL = (“technology-assisted therapy”)) AND (ALL = (stroke) OR ALL = (“brain injury”) OR ALL = (“traumatic brain injury”) OR ALL = (“spinal cord injury”) OR ALL = (“multiple sclerosis”) OR ALL = (“Parkinson’s disease”) OR ALL = (parkinson) OR ALL = (“cerebral palsy”) OR ALL = (“neurological disorders”)) AND (AB = (“cost minimization”) OR AB = (“cost effectiveness”) OR AB = (“cost utility”) OR AB = (“cost benefit”) OR TI = (cost) OR AB = (“cost-analysis”) OR ALL = (“economic analysis”) OR ALL = (“economic evaluation”))	171

**Table 3:** Clinical characteristics of the included studies.

Study	Location	Disease	Setting	Sample	Male/Female	Age, Mean ± SD	Technology/Body Part	Total Training Hours (or Protocol)	Type of Economic Cost Study
Hesse et al., 2014 [18]	Germany	Stroke	Clinical setting	50 subacute stroke patients: *n* = 25 (robot-assisted group therapy + individual arm therapy) *n* = 25 (two sessions of individual arm therapy)	13/12 15/10	71.4 ± 15.5 69.7 ± 16.6	Robot/upper limb	Experimental group: 30 min of robot therapy + 30 min of individual arm therapy per workday for four weeks; supervised by a therapy assistant Control group: 2 × 30 min of individual arm therapy per workday for four weeks; supervised by an experienced therapist	Cost minimization Cost benefit Description of costs
Bustamante et al., 2016 [19]	Mexico	Stroke	Clinical setting	20 chronic stroke patients: *n* = 10 (traditional therapy) *n* = 10 (Robot Gym)	4/6 3/7	64.1 ± 8.38 44.1 ± 12.55	Robot/upper limb and lower limb	24 two-hour therapy sessions over a period of 6 to 8 weeks for all study subjects	Cost-effectiveness Description of costs
McCabe et al., 2015 [20]	USA	Stroke	Clinical setting	35 chronic stroke patients: *n* = 11 (motor learning) *n* = 12 (robot + motor learning) *n* = 12 (FES + motor learning)	6/5 10/2 7/5	NR NR NR	Robot/upper limb	5 days/week for 5 h/day (60 sessions) for all groups	Cost-effectiveness Description of costs
Wagner et al., 2011 [21]	USA	Stroke	Clinical setting	127 chronic stroke patients: *n* = 49 (robot) *n* = 50 (intensive comparison therapy) *n* = 28 (usual care)	47/2 48/2 27/1	66 ± 11 64 ± 11 63 ± 12	Robot/upper limb	Three 1 h sessions per week for 12 weeks, 36 sessions in total	Cost minimization Cost utility Description of costs
Masiero et al., 2014 [22]	Italy	Stroke	Clinical setting	35 acute stroke patients: *n* = 17 (robot) *n* = 18 (robot plus exercise with unimpaired upper limb) 21 acute stroke patients: *n* = 11 (robot) *n* = 10 (usual care) 30 acute stroke patients: *n* = 14 (robot + usual care) *n* = 16 (usual care)	10/7 11/7 9/2 7/3 10/4 10/6	63.4 ± 11.8 68.8 ± 10.5 72.4 ± 7.1 75.5 ± 4.8 65.6 ± 9.2 66.83 ± 7.9	Robot/upper limb	Two daily sessions of 25 min each with robot, for 5 days per week. The two protocols were compared (in terms of number of weeks).	Cost-effectiveness Description of costs
Housley et al., 2016 [23]	USA	Stroke	Home	20 chronic stroke patients: *n* = 10 (upper limb robot) *n* = 10 (lower limb robot)	9/1 10/0	63.4 ± 9.1 70.6 ± 12.7	Robot/upper limb and lower limb	Each person was instructed to start at lower daily activity levels (one hour), progressing to the standard two-hour therapy dosage within the first week, which was continued for the three-month study duration. Due to the scheduling flexibility of the robotic device, participants were able to complete the two hours of daily prescribed robotic rehabilitation in any permutation.	Cost utility Cost benefit Description of costs
Chan et al., 2022 [24]	China	Stroke	Clinical setting	NR	NR	NR	Robot/lower limb	NR	Cost minimization Cost benefit Description of costs
Fernández-García et al., 2021 [25]	UK	Stroke	Clinical setting	770 acute and chronic stroke patients: *n* = 257 (robot-assisted training plus usual care) *n* = 259 (EULT programme plus usual care) *n* = 254 (usual care)	156/101 159/100 153/101	59.9 ± 13.5 59.4 ± 14.3 62.5 ± 12.5	Robot/upper limb	Robot-assisted training: 45 min per day, three days per week for 12 weeks, in addition to usual care EULT: 45 min per day, 3 days per week for 12 weeks, in addition to usual care Usual care: 12-week period	Cost minimization Cost-effectiveness Cost utility Description of costs
Rodgers et al., 2020 [26]	UK	Stroke	Clinical setting	770 acute and chronic stroke patients: *n* = 257 (robot-assisted training plus usual care) *n* = 259 (EULT programme plus usual care) *n* = 254 (usual care)	156/101 159/100 153/101	59.9 ± 13.5 59.4 ± 14.3 62.5 ± 12.5	Robot/upper limb	Robot-assisted training: 45 min per day, three days per week for 12 weeks, in addition to usual care EULT: 45 min per day, 3 days per week for 12 weeks, in addition to usual care Usual care: 12-week period	Cost minimization Cost effectiveness Cost utility Description of costs
Pinto et al., 2023 [27]	USA	Spinal cord injury	Clinical setting	99 SCI patients: *n* = 67 SCI patients (conventional training) *n* = 32 SCI patients (overground robotic training)	46/21 20/12	42 ± 16 33 ± 13	Robot/lower limb	Authors declared that “training was not standardized as is typical in practice-based evidence design”. Around 60 min for robotic intervention versus 45 min for the overground group. Donning and doffing of the robotic exoskeleton added non-therapeutic time (potentially 40 min). The overground robotic training group had greater training times.	Cost utility Description of costs
Lloréns et al., 2015 [28]	Spain	Stroke	Clinical setting versus at home	30 chronic stroke patients: *n* = 15 (in-clinic rehabilitation using VR) *n* = 15 (at-home intervention using VR)	10/5 7/8	55.47 ± 9.63 55.60 ± 7.29	Virtual reality/lower limb	Twenty 45 min training sessions conducted 3 times a week for 8 weeks. Both groups received conventional physical therapy in a clinic.	Cost minimization Description of costs
Islam et al., 2019 [29]	Denmark, Norway and Belgium	Stroke	Clinical setting	102 subacute stroke patients: *n* = 50 (VR training) *n* = 52 (conventional training)	NR NR	NR NR	Virtual reality/upper limb	Sixteen 60 min sessions over 4 weeks	Cost minimization Cost benefit Description of costs
Adie et al., 2017 [30]	UK	Stroke	Home	235 subacute stroke patients: *n* = 117 (Wii ^®^ intervention) *n* = 118 (arm exercises at home)	66/51 65/53	66.8 ± 14.6 68.0 ± 11.9	Virtual reality/upper limb	Daily sessions for six weeks	Cost minimization Cost benefit Description of costs
Thomas et al., 2017 [31]	UK	Multiple sclerosis	Home	30 MS patients (EDSS NR): *n* = 15 (Nintendo Wii + usual care) *n* = 15 (usual care)	1/14 2/13	50.9 ± 8.08 47.6 ± 9.26	Virtual reality/upper and lower limb	12 months and 6 months of treatment for each group, respectively. Rest of the protocol data were NR.	Cost minimization Description of costs
Farr et al., 2021 [32]	UK	Cerebral palsy	Home	30 cerebral palsy patients (GMFCS levels I–II): *n* = 15 (supervised VR group) *n* = 15 (unsupervised VR group)	12/3 10/5	27% <11 years 27% >11 years	Virtual reality/lower limb	12 weeks of treatment (rest of the protocol data were NR).	Cost minimization

EDSS: Expanded Disability Status Scale; GMFCS: Gross Motor Function Classification System; *n*: sample size; NR: not recorded; SD: standard deviation; SCI: spinal cord injury; VR: virtual reality.

**Table 4:** Economic characteristics.

Study	Currency	Cost Data (CG)	Cost Data (EG)	Trial Duration	Authors’ Economic Conclusion
Hesse et al., 2014 [18]	EUR	The experienced therapist in the control group treated 3825 patients per year; the total costs (salary, 10% overhead) of the individual arm therapy were EUR 38,500, i.e., one treatment cost EUR 10.00. Thus, the difference in actual costs for the employer was EUR 5.85 per session.	The net investment costs for the devices (EU list prices) plus a 25% overhead (for maintenance, energy, consumables) were EUR 48,000, to be deducted within four years resulting in an annual cost of EUR 12,000. The annual gross salary of the assistant therapist was EUR 25,000, and it was EUR 35,000 for the experienced therapist. The assistant therapist in the experimental group treated 8925 patients per year, thus the total costs (device, overhead, salary) of the robot-assisted group therapy were EUR 37,000, i.e., one treatment cost EUR 4.15.	1 month	Robot-assisted group therapy (comprising **Bi-ManuTrack^®^, RehaDigit^®^, Reha-Slide^®^ and Reha-Slide duo^®^**, all considered end-effector robot devices) + individual arm therapy were as effective as a double session of individual arm therapy in subacute stroke patients. Robot-assisted group therapy is probably more cost-efficient than individual arm therapy. The robot-assisted group therapy, supervised by an assistant therapist, cost less.
Bustamante et al., 2016 [19]	MXN	The salary of a rehabilitation specialized therapist in the Mexican public health institution was reported to be around MXN 235,344 (USD 19,612) per year. Traditional therapy, which consisted of the time-matched standard of care where patients received 2 h of therapy, was estimated to have a therapy cost of MXN 230.52 (USD 19.21) per session.	The full cost of the robotic equipment (adding transportation and importation costs) was MXN 432,592.4, to be settled within 2 years with annual payments of MXN 216,296 (approximately USD 18,024 at that time). For the robotic therapy group, the therapy cost would be MXN 83.90 (USD 6.99) within the first 2 year and after this period of time, the net cost of the equipment will be liquidated and only the percentage reserved for maintenance will remain, reducing the estimated cost for robotic therapy to MXN 51.48 (USD 4.29) per session with a 2 h therapy session per patient.	2 months	**Robot Gym** (Theradrive system^®^ + Ness for upper extremity^®^ + Ness for lower extremity^®^ + Motomed Viva 2^®^ for upper extremities + Motomed Viva 2^®^ for lower extremities + Captain’s Log Brain-trainer^®^; all the robots considered were end-effector robot devices) enhanced functionality in the upper-extremity tests similarly to patients in the control group. In the lower-extremity tests, the EG showed a greater improvement compared to those subjected to traditional therapy. Robot Gym could be a more cost- and labour-efficient option for countries with scarce clinical resources and funding.
McCabe et al., 2015 [20]	USD	Therapist cost was USD 98,000, which is the annual salary for an experienced therapist in Ohio where the study was conducted FES cost for a 4-channel tabletop and 2-channel portable system was USD 4000, with a 5-year equipment life Motor learning approach treatment: USD 4570 FES plus motor learning approach: USD 4604	Shoulder/elbow clinical level robot with 5-year life cost: USD 89,000; annual robot warranty and maintenance: USD 8000 Robotics plus motor learning approach: USD 5686	3 months	Severely impaired stroke survivors with persistent (>1 year) upper-extremity dysfunction can make clinically and statistically significant gains in coordination and functional task performance in response to treatment with **InMotion2^®^ Shoulder-Elbow Robot** (end-effector robot device) plus a motor learning approach, FES plus motor learning approach, and motor learning approach alone in an intensive and long-duration intervention; no group differences were found. The motor learning approach alone protocol was less expensive than the robotics plus motor learning approach protocol (by USD 1116) and the FES plus motor learning approach protocol (by USD 34). Therefore, if a cost differential of approximately USD 1000 per patient is considered important, the FES plus motor learning approach protocol and/or the motor learning approach alone protocol would be preferable.
Wagner et al., 2011 [21]	USD	Cost per session of the intensive comparison therapy: USD 218 Average cost: USD 7382	Cost per session of the robot training: USD 140. Average cost: USD 5152	3 months	The average cost of delivering robot therapy (**MIT-Manus**^®^, considered as an end-effector robot device) and intensive comparison therapy was USD 5152 and USD 7382, respectively, and both were significantly more expensive than usual care alone (no additional intervention costs). The added cost of delivering robot or intensive comparison therapy was recuperated by lower healthcare use costs compared with those in the usual care group. The changes in quality of life were modest and not statistically different. Cost data were analysed at 36 weeks post-randomization.
Masiero et al., 2014 [22]	EUR	Hourly/year physiotherapist cost: EUR 18,773.	Hourly/year cost (robot + therapist; ratio 1 robot/therapist): EUR 25,119 Hourly/year cost during (robot + therapist; ratio 3 robots/therapist): EUR 12,604	1 month versus 1 month and 1 week	By comparing several **NeReBot**^®^ (end-effector robot device) treatment protocols, comprising different combinations of robotic and non-robotic exercises, the authors showed that robotic technology can be a valuable and economically sustainable aid in the management of post-stroke patient rehabilitation.
Housley et al., 2016 [23]	USD	Projected outpatient therapy based on three 1 h weekly physical therapy sessions for 90 days: USD 3619.95.	Monthly costs of home-based robot-assisted therapy: USD 1268.07	3 months	Home-based, robotic therapy (**Hand and Foot Mentor**^®^, considered a hybrid robot device) reduced costs, while expanding access to a rehabilitation modality for people who would not otherwise have received care. The analysis revealed an average of USD 2352 (64.97%) in savings compared to clinic-based therapy per stroke survivor. Further, the inclusion of home-based telerehabilitation leads to a return of approximately USD 2.85 for therapy on every dollar spent by the health system.
Chan et al., 2022 [24]	HKD	Therapist salary: HKD 63,000 Total hourly cost (therapists): HKD 269.23	Total machine cost: HKD 1,759,200.00 Total hourly cost (robot): HKD 175.92	NR	**ROBERT^®^** (end-effector robot device) was better than physical therapy in performing repetitive exercises for lower limbs. The physiotherapist’s time can be saved when the robot is being used. The cost analysis result showed that employing **ROBERT^®^** is less costly than the equivalent performed by a physiotherapist. Its cost benefit was HKD 175.92/one eff. unit, whereas that of physical therapy is HKD 269.23/one eff. unit. Although the capital cost of the robotic system was high, its average hourly operating cost was just one-tenth of the cost for one specialty outpatient session in a hospital.
Fernández-García et al., 2021 [25]	GBP	Usual care: GBP 3785 EULT: GBP 4451	Robot-assisted training: GBP 5387	3 months	The cost-effectiveness analysis suggested that neither robot-assisted training with **MIT-Manus robotic gym (InMotion**^®^ **commercial version,** considered an end-effector robot device) nor EULT, as delivered in this trial, were likely to be cost-effective at any of the cost-per-QALY thresholds considered. At 6 months, on average, usual care was the least costly option (GBP 3785), followed by EULT (GBP 4451), with robot-assisted training being the most expensive (GBP 5387). The mean difference in total costs between the usual care and robot-assisted training groups (GBP 1601) was statistically significant (*p* < 0.001). The mean QALY was highest for the EULT group (0.23) but there was no evidence of a difference (*p* = 0.995) between the robot-assisted training (0.21) and usual care groups (0.21). Cost-effectiveness acceptability curves showed that robot-assisted training was unlikely to be cost-effective and that EULT had a 19% chance of being cost-effective at the GBP 20 000 WTP threshold. Usual care was most likely to be cost-effective at all the WTP values considered in the analysis.
Rodgers et al., 2020 [26]	GBP	Usual care: GBP 3785 EULT: GBP 4451	Robot-assisted training: GBP 5387	3 months	The RATULS trial did not find evidence that a robot-assisted training programme using the **MIT-Manus robotic gym (InMotion**^®^ **commercial version,** considered an end-effector robot device), as implemented in this trial, improved upper limb function following a stroke when compared with an EULT programme based on goal-orientated repetitive functional task practice at the same frequency and duration, or with usual care. Neither robot-assisted training nor the EULT programme as provided in the RATULS trial (1:1 patient-to-therapist ratio) were cost-effective at the current UK WTP per QALY (GBP 20,000–30,000).
Pinto et al., 2023 [27]	USD	Conventional training cost: USD 1758 Litegait overground training system: USD 0.47/session for rehabilitation hospital purchasing department. Body weight-supported treadmill and harness system: USD 6.86/session Rehabilitation hospital-quality treadmill: USD 35,000 + annual maintenance contract (USD 8500)	Robotic training cost: USD 3952 Overground exoskeleton device: USD 18.36/session Capital cost of robot (purchase price): USD 150,000 + annual maintenance contract (USD 10,000) Stationary robotic system: USD 38.95/session (USD 350,000 + annual maintenance contract (USD 15,000)) Track-based overground training and harness system: USD 7.52/session (USD 225,000 + annual maintenance contract (USD 7500))	NR	The most cost-effective locomotor training strategy for people with an SCI differed depending on injury completeness: conventional overground training was more effective and cost less than robotic therapy (**type of robot/s used not reported**) for people with an incomplete SCI. Overground robotic training was more effective and cost more than conventional training for people with a complete SCI. Costs were lower for conventional training (USD 1758) versus overground robotic training (USD 3952) and lower for those with an incomplete versus complete injury. The incremental cost utility ratio for overground robotic training for people with a complete spinal cord injury was USD 12,353/QALY.
Lloréns et al., 2015 [28]	USD	The overall expense for one participant belonging to the in-clinic programme was USD 1490.23	The home-based programme required an estimated expenditure of USD 800 to acquire the hardware needed for the VR system	2 months	VR-based telerehabilitation interventions can promote the reacquisition of locomotor skills associated with balance in the same way as in-clinic interventions, both complemented by a conventional therapy programme. The telerehabilitation intervention can involve savings (mainly derived from transportation services) compared with the in-clinic intervention. Both treatment modalities used a computer/laptop, **Kinect**^®^ (semi- immersive virtual reality system) and Internet access. The cost of one hour of physical therapy was USD 21.85. The difference between the two interventions was USD 654.72 (in favour of the telerehabilitation intervention). Beyond human resources, the most influential factor was the travel expenses (USD 1308.11), which represented 87.77% of the total cost of the in-clinic intervention.
Islam et al., 2019 [29]	USD	The average monthly take-home salary of an experienced physiotherapist in Norway is approximately USD 3224. Hence, the average hourly wage is about USD 21.5 (USD 35.72, including the income tax and social security contribution costs for both the employee and the employer).	The price of one YouGrabber Basic system is equivalent to USD 7544 including VAT.	1 month	The YouGrabber^®^ system (now called **Bi-Manu-Trainer**^®^, is considered a semi-immersive virtual reality system) was used. In the VIRTUES trial, no cost savings in favour of VR were found. Additional upper-extremity VR training was equally as effective as additional conventional therapy in the subacute phase after stroke.
Adie et al., 2017 [30]	GBP	Arm exercises group cost: GBP 730	Wii^®^ group cost: GBP 1106	1 month and 2 weeks	**Wii^®^** (non-immersive virtual reality system) was not superior to arm exercises in home-based rehabilitation for stroke survivors with arm weakness. **Wii^®^** was well tolerated but more expensive than arm exercises.
Thomas et al., 2017 [31]	GBP	Using an estimated cost of GBP 32 per hour for a hospital physiotherapist equates to a per participant cost of the intervention of GBP 384 for physiotherapy time.	The equipment cost (Nintendo Wii^®^ console plus peripherals and software) was approximately GBP 300 per unit. The mean cost of delivering Mii-vitaliSe was GBP 684 per person.	12 months and 6 months, for each group	**A Nintendo Wii**^®^ system, considered a non-immersive virtual reality system (Wii Fit Plus^®^, Wii Sports^®^ and Wii Sports Resort^®^ along with the Wii Balance Board (and non-slip cover), two Wii remote controls, two Nunchuk controls, battery and remote control chargers and spare rechargeable batteries), was used. The mean time per participant spent by the physiotherapists delivering Mii-vitaliSe was 12 hours with approximately half of this time involving face-to-face contact.
Farr et al., 2021 [32]	GBP	NR	The cost of a therapist’s time over the 12-week intervention was GBP 20.10 per child in the supported group (Nintendo Wii Fit^®^). This is based on an hourly rate for a band 5 physiotherapist (AfC specialist level) of GBP 37. The physiotherapists in this study, however, were band 7 (advanced/team leader) and 8 (principal/consultant). Costs at these higher levels would be around GBP 30 or GBP 40 per child, respectively.	3 months	Therapeutic use of **Nintendo Wii**^®^**,** considered a non-immersive virtual reality system, with the Wii Balance Board ^®^ and Wii Fit^®^ in-home was inexpensive and acceptable in short periods of around six weeks.

CG: control group; EG: experimental group; EU: European Union; EULT: enhanced upper limb therapy; FES: functional electrical stimulation; SCI: spinal cord injury; WTP: willingness to pay; VAT: Value-Added Tax; VR: virtual reality.

**Table 5:** Methodological quality of the included studies (using Joanna Briggs Institute for economic evaluation instrument).

Study	Q1	Q2	Q3	Q4	Q5	Q6	Q7	Q8	Q9	Q10	Q11	Total
**Robotic devices**												
Hesse et al., 2014 [18]	Yes	Yes	Yes	Yes	Yes	Yes	No	No	No	No	No	6/11
Bustamante et al., 2016 [19]	Yes	Yes	Yes	Yes	Yes	Yes	No	No	No	No	No	6/11
McCabe et al., 2015 [20]	No	Yes	Yes	Yes	Yes	Yes	No	No	No	No	Yes	6/11
Wagner et al., 2011 [21]	Yes	Yes	Yes	Yes	Yes	Yes	Yes	Yes	Yes	Yes	Yes	11/11
Masiero et al., 2014 [22]	Yes	No	Yes	Yes	Yes	Yes	Yes	No	No	No	Yes	7/11
Housley et al. 2016 [23]	Yes	No	No	Yes	Yes	Yes	No	No	No	Yes	No	5/11
Chan et al., 2022 [24]	Yes	No	No	No	No	No	No	Yes	No	No	No	2/11
Fernández-García et al., 2021 [25]	Yes	No	Yes	Yes	Yes	Yes	Yes	Yes	Yes	No	Yes	9/11
Rodgers et al., 2020 [26]	Yes	No	Yes	Yes	Yes	Yes	Yes	Yes	Yes	Yes	Yes	10/11
Pinto et al., 2023 [27]	Yes	Yes	Yes	Yes	Yes	Yes	No	Yes	No	No	No	7/11
**Virtual reality devices**												
Lloréns et al., 2015 [28]	Yes	Yes	Yes	Yes	Yes	Yes	No	No	No	Yes	Yes	8/11
Islam et al., 2019 [29]	Yes	Yes	Yes	Yes	Yes	Yes	Yes	No	No	No	No	7/11
Adie et al., 2017 [30]	Yes	Yes	Yes	Yes	Yes	Yes	No	Yes	No	Yes	Yes	9/11
Thomas et al., 2017 [31]	Yes	Yes	Yes	No	No	Yes	No	No	No	No	No	4/11
Farr et al., 2021 [32]	Yes	Yes	Yes	No	Yes	Yes	No	No	No	No	No	5/11
**Total %**	93.33	66.66	86.66	86.66	86.66	93.33	33.33	40	20	33.33	46.66	

JBI critical appraisal checklist for economic evaluations: Q1. Is there a well-defined question? Q2. Is there a comprehensive description of alternatives? Q3. Are all important and relevant costs and outcomes for each alternative identified? Q4. Has clinical effectiveness been established? Q5. Are costs and outcomes measured accurately? Q6. Are costs and outcomes valued credibly? Q7. Are costs and outcomes adjusted for differential timing? Q8. Is there an incremental analysis of costs and consequences? Q9. Were sensitivity analyses conducted to investigate uncertainty in estimates of cost or consequences? Q10. Do study results include all issues of concern to users? Q11. Are the results generalizable to the setting of interest in the review?

## Data Availability

Data are contained within the article.

## Abbreviations

PRISMA	Preferred Reporting Items for Systematic Reviews and Meta-Analyses
WOS	Web of Science
CHEERS	Consolidated Health Economic Guide Reporting standards
HMDs	Head-mounted displays
CAVE	Cave-Assisted Virtual Environments
